# Effects of IFN-β1a and IFN-β1b treatment on the expression of cytokines, inducible NOS (NOS type II), and myelin proteins in animal model of multiple sclerosis

**DOI:** 10.1007/s00005-017-0458-6

**Published:** 2017-03-15

**Authors:** Natalia Lubina-Dąbrowska, Adam Stepień, Grzegorz Sulkowski, Beata Dąbrowska-Bouta, Józef Langfort, Małgorzata Chalimoniuk

**Affiliations:** 1 Neurology Clinic, Military Institute of Medicine, Warsaw, Poland; 2 Laboratory of Pathoneurochemistry, Department of Neurochemistry, Mossakowski Medical Research Centre Polish Academy of Sciences, Warsaw, Poland; 3 Department of Experimental Pharmacology, Mossakowski Medical Research Centre, Polish Academy of Sciences, Warsaw, Poland; 4 Department of Sports Training, The Jerzy Kukuczka Academy of Physical Education, Katowice, Poland; 5 Department of Cellular Signalling, Mossakowski Medical Research Centre, Polish Academy of Sciences, Pawińskiego 5, 02-106 Warsaw, Poland

**Keywords:** Interferon beta, Cytokines, Inducible nitric oxide synthase, EAE

## Abstract

The aim of this study was to investigate the effects of interferon (IFN)-β1a and IFN-β1b treatment on inflammatory factors and myelin protein levels in the brain cortex of the Lewis rat experimental autoimmune encephalomyelitis (EAE), animal model of multiple sclerosis. To induce EAE, rat were immunized with inoculums containing spinal cord guinea pig homogenized in phosphate-buffered saline and emulsified in Freund’s complete adjuvant containing 110 µg of the appropriate antigen in 100 µl of an emulsion and additionally 4-mg/ml *Mycobacterium tuberculosis* (H37Ra). The rats were treated three times per week with subcutaneous applications of 300,000 units IFN-β1a or IFN-β1b. The treatments were started 8 days prior to immunization and continued until day 14 after immunization. The rats were killed on the 14th day of the experiment. EAE induced dramatic increase in interleukin (IL)-1β, IL-6, and tumor necrosis factor (TNF)-concentrations and inducible nitric oxide synthase (iNOS) expression in the brain, which closely corresponded to the course of neurological symptoms and the loss of weight. Both IFN-β1b and IFN-β1a treatments inhibited the pro-inflammatory cytokines (IL-6, IL-1β, TNF-α and IFN-γ), decreased the activation of astrocytes, increased the myelin protein level in the brain cortex, and improved the neurological status of EAE rats by different mechanisms; IFN-β1a reduced iNOS expression, at least in part, by the enhancement of IL-10, while IFN-β1b diminished IL-10 concentration and did not decrease EAE-induced iNOS expression.

## Introduction

Multiple sclerosis (MS) is a chronic, inflammatory neurodegenerative disease, which is characterized by demyelination and remyelination, and neuronal damage (Holz et al. 2000; Stadelmann 2011; Stüve and Oksenberg 2010), with onset of disease typically occurring between the ages of 20 and 40 years (Sospedra and Martin 2005). Demyelinating lesions are often found in the white matter of the brain stem, the spinal cord, and the cerebellum (Compston and Coles 2008). After a course of the relapsing-remitting phase of the disease, most MS patients enter a phase characterized by progressive neurodegeneration associated with an irreversible variety of physical disabilities (Ireland and Monson 2011; Lucchinetti et al. 2000; Trapp et al. 1999). Pathological and magnetic resonance imaging studies indicate that axonal damage predominantly develops in the early stage of MS as a consequence of inflammatory process (Bendfeldt et al. 2009; Comi 2000), which leads to the most numerous (~85% of cases) relapsing-remitting form of the disease (Weiner 2008).

A considerable infiltration by macrophages, monocytes, and lymphocytes into central nervous system during MS induces secretion of many activated microglia and astrocytes pro-inflammatory cytokines, including interleukin (IL)-1β, tumor necrosis factor (TNF)-α and IL-6 (Aguzzi et al. 2013; Hartung et al. 1995), which are involved in the production of oxidative radicals (Merrill and Benveniste 1996) and expression of the inducible nitric oxide synthase (iNOS) (Willenborg et al. 1999). Elevated levels of aforementioned pro-inflammatory cytokines in the plasma, cerebrospinal cord and brain cortex were found in patients with MS (Giovannoni and Thorpe 2001; Navikas et al. 1996a, b; Rieckmann et al. 1995; Sharief and Hentges 1991) and a positive correlation was found between the levels and the disease’s activity and severity (Navikas et al. 1996b; Rieckmann 1995; Sharief and Hentges 1991). A similar effect, i.e. elevation of pro-inflammatory cytokines (including IFN-γ, IL-1β, TNF-α, and IL-6) was observed in experimental autoimmune encephalomyelitis (EAE), an animal model of MS (Schneider et al. 2009; Sulkowski et al. 2013; Tanuma et al. 1997). The increase in the production of cytokines with pro-inflammatory potential is generally accompanied by concomitant increase in the production of cytokines with anti-inflammatory/immunoregulatory properties, among which TGF-β and IL-10 play a dominant role (Imitola et al. 2005). Thus, disordered balance between pro- and anti-inflammatory mediators may lead to induction and progression of MS/EAE (Brosnan and Raine 1996).

There are two structural forms of IFN-β, i.e. IFN-β1b and IFN-β1a, that are used therapeutically. They demonstrate a good efficacy in long-term relapsing-remitting of MS (RR-MS) therapy (Bendfeldt et al. 2010; Stępień et al. 2013), and also to some extent in the treatment of secondary progressive MS (European Study Group on Interferon β-1b in Secondary Progressive MS 1998). However, the biological activities of IFN-β1b and IFN-β1a are not the same. It is known that the IFN-β1a has a higher biological potency in its antiviral properties (Antonetti et al. 2002). Following this notion, these two IFN-β variants altered diversely the plasma cytokine profile of RR-MS patients following 3-year therapy, despite similar improvement of neurological status and marked reduction of the annual relapse rate in a majority of RR-MS patients with mild to moderate disability (Stępień et al. 2013).

Growing evidence demonstrates that the inflammatory process is most active at the beginning of MS (Comi et al. 2001) and takes part in degeneration of the myelin sheath of nerve cells. As one of the plausible modes of IFN-β action in responsive patients is anti-inflammatory effect (Graber et al. 2007; Liu et al. 2010; Ransohoff et al. 1991; Rio and; Montalban 2005; Salama et al. 2003), the improved results of IFN-β therapy could be especially expected from early treatment of MS, when the inflammatory process initiates. This issue is poorly investigated in the clinical practice and relatively little is known about inflammatory process leading to demyelination as well as efficacy of IFN-β1b and IFN-β1a treatment on this process at the beginning of MS, when disease in most patients may be ongoing subclinically. To better recognize this issue we have investigated the effects of IFN-β1a and IFN-β1b monotherapies on selected serum cytokines and nitrite levels in the early phased of EAE rats. This study was also aimed to investigate the influence of IFN-β1a and IFN-β1b treatment on the iNOS and myelin protein levels (indicated by MOG and CNP-ase levels) and their possible adjustment by regulation of selected pro- and anti-inflammatory cytokines in the cerebral cortex in rats subjected to EAE.

## Materials and methods

### Induction of EAE in animals

Eight-week-old, female Lewis rats (183 ± 10 g) were supplied by the animal house of the Mossakowski Medical Research Centre, Polish Academy of Sciences. All procedures involving rodent care and experimentation were carried out in accordance with the European Communities Council Directive (86/609/EEC) for the Care and Use of Laboratory Animals. All protocols were approved by the 4th Local Ethics Committee for Animal Experiments, National Medicines Institute, Warsaw, Poland. Rats were housed in a temperature-controlled room with a 12-h light/dark cycle and free access to water and food, including the Ssniff^®^ R-2 complete diet for rat breeding (Ssniff Spezialdoten GmbH, Soest, Germany).

To induce EAE, female Lewis rats were immunized with inoculums, which contained spinal cord guinea pig homogenized in phosphate-buffered saline, and emulsified in Freund’s complete adjuvant, which contained 110 µg of the appropriate antigen in 100 µl of emulsion and 4-mg/ml *Mycobacterium tuberculosis* (H37Ra) (Kerschensteiner et al. 2004; Meyer et al. 2001). The animals were observed daily and monitored for neurological deficits with clinical severity scores and weight. The clinical scores of EAE were assigned according to the following criteria—0: asymptomatic; 1: complete loss of tail tone; 2: hind limb paraplegia; 3: complete hind limb paralysis; 4: hind limb paralysis with forelimb involvement; and 5: moribund/dead (Kerschensteiner et al. 2004; Meyer et al. 2001). Four different experimental groups of animals were used: control, EAE, EAE treated with IFN-β1a, and EAE treated with IFN-β1b.

### Administration of IFN-β1a or IFN-β1b to rats with EAE

The treatments of IFN-β1a or IFN-β1b were started 8 days after immunization and continued until day 14 after immunization (Wender et al. 2001). The rats with EAE were treated three times per week with subcutaneous applications of 300,000 units of IFN-β1a (Biogen IDEC LTD, Berkshire, UK) or IFN-β1b (Bayer Schering Pharma, Berlin, Germany). The rats were euthanized on the 14th day of the experiment.

### Real-time reverse transcriptase-polymerase chain reaction

Total RNA was extracted from the brain cortex (gray and white matter) using TRI Reagent (Sigma, St. Louis, MO, USA), and 2-µg RNA were reverse-transcribed (RT) using random primers and AMV reverse transcriptase (Life Technology, Carlsbad, CA, USA). The RT conditions included: reverse transcription at 42 °C for 45 min, denaturation at 94 °C for 30 s. For quantitative reverse transcriptase-polymerase chain reaction (RT-PCR) analysis, the TaqMan technology was employed. Rat cytokines (IL-1β-Rn00580432_m1; IL-6-Rn01410330_m1; TNF-α-Rn00563254_m1; and IFN-γ-Rn00594078_m1, IL-10-Rn00563409_m1), the receptors IL-1r1-Rn00565482_m1 and IL-1r2-Rn00588589_m1, iNOS-Rn00561646_m1) specific primers, and the probes were obtained from Life Technology (Carlsbad, CA, USA). To normalize the expression of the cytokines, the receptors IL-1r1 and IL-1r2, and iNOS mRNA, the actin levels (endogenous controls) were determined using TaqMan assay reagents (Applied Biosystems, Carlsbad, CA, USA). Real time-PCR was conducted with an ABI 7500 system (Applied Biosystems, Carlsbad, CA, USA) using 5 µl of RT product, a TaqMan PCR Master Mix, primers, and a TaqMan probe (Life Technology, Carlsbad, CA, USA) in a total volume of 20 μl. The cycle conditions of the PCR were as follows: initial denaturation at 95 °C for 10 min, 50 cycles of 95 °C for 15 s, and 60 °C for 1 min. Each sample was analyzed in triplicate. The relative expression levels of the cytokines were calculated using the standard curve method and were normalized to actin.

### Gel electrophoresis and western blotting for IL-1β, IL-6, IFN-γ, TNF-α, iNOS, MOG, and CNPase

Brain cortex homogenate aliquots (40-µg protein) were mixed with an equal volume of sample buffer (62.5-mM Tris–HCl, 2% SDS, 100-mM DTT, 20% glycerol, and 0.2% bromophenol blue, pH 6.8) and heated for 5 min at 95 °C, electrophoresed on 10% polyacrylamide gel (Laemmli 1970). They were then electrotransferred to nitrocellulose membranes and blocked with a 5% non-fat milk powder solution in Tris-buffered saline containing 0.05% Tween 20 (TBS-T) for 1 h at 37 °C. Then, the membranes were incubated with polyclonal anti-IL-6, anti-IL-1β, anti-TNF-α, anti-iNOS, anti-MOG, and anti-CNPase antibodies (diluted as described in Table 1) overnight at 4 °C. Next, the membranes were incubated with the relevant secondary antibody conjugated with horseradish peroxidase (diluted in TBS-T containing 5% non-fat milk; see Table 1) for 1 h at room temperature. The protein bands were visualized on an autoradiographic Hyperfilm-Kodak (Sigma–Aldrich, St. Louis, MO, USA) using an ECL kit (Thermo Fisher Scientific Inc. Rockford, IL, USA). The cytokine, iNOS, MOG, and CNPase bands were quantified using a NucleoVision apparatus and the GelExpert 4.0 software (Nucle Tech Corporation, San Matea, CA, USA).

**Table 1:** Description of primary and secondary antibody used in this paper

Name of protein	Primary antibody; Cat. No.; dilution, company	Second antibody; dilution, company
GFAP	Polyclonal anti-GFAP antibody; No. G9269; 1:250 in TBS-T; Sigma-Aldrich, St. Louis, MO, USA	Anti-rabbit-HRP; 1:8000 in TBS-T with 5% skim milk; Sigma-Aldrich, St. Louis, MO, USA
IL-1β	Polyclonal anti-IL-1β antibody; No. I4893; 1:500 in TBS-T, Sigma-Aldrich, St. Louis, MO, USA	Anti-rabbit-HRP; 1:8000 in TBS-T with 5% skim milk; Sigma-Aldrich, St. Louis, MO, USA
IL-6	Polyclonal anti-IL-6 antibody; No. I3393; 1:500 in TBS-T; Sigma-Aldrich, St. Louis, MO, USA	Anti-rabbit-HRP; 1:8000 in TBS-T with 5% skim milk; Sigma-Aldrich, St. Louis, MO, USA
IFN-γ	Polyclonal anti-IFN-γ antibody; No. I9141; 1:500 in TBS-T, Sigma-Aldrich, St. Louis, MO, USA	Anti-rabbit-HRP; 1:8000 in TBS-T with 5% skim milk; Sigma-Aldrich, St. Louis, MO, USA
TNF-α	Polyclonal anti-TNF-α antibody; No. T3198; 1:500 in TBS-T; Sigma-Aldrich, St. Louis, MO, USA	Anti-rabbit-HRP; 1:8000 in TBS-T with 5% skim milk; Sigma-Aldrich, St. Louis, MO, USA
CNP-ase	Monoclonal CNP-ase antibody; No. C5922; 1:300 in TBS-T; Sigma-Aldrich, St. Louis, MO, USA	Anty-mouseHRP, 1:2000 in TBS-T with 5% skim milk; Sigma-Aldrich, St. Louis, MO, USA
MOG	Polyclonal anti-MOG antibody; No. M0695; 1:1000 in TBS-T, Sigma- Polycolnal anty-iNOS antibody; 1:25,000 in TBS-T; BD Biosciences, Aldrich, St. Louis, MO, USA	Anty-goat-HRP; 1:50,000 in TBS-T with 5% skim milk; Sigma-Aldrich, St. Louis, MO, USA
iNOS	Mouse monoclonal anti-iNOS antibody; No. 350; 1:500; Becton-Dickinson, Ermbodegem, Belgium	Anty-mouse-HRP; 1:1000 in TBS-T with 5% skim milk; Becton-Dickinson, USA
GAPDH	Polyclonal anti-GAPDH antibody; No. G9545; 1:40,000 in TBS-T; Sigma- Aldrich, St. Louis, MO, USA	Anty-goat-HRP; 1:5000 in TBS-T with 5% skim milk; Sigma-Aldrich, St. Louis, MO, USA

*HRP* horseradish peroxidase

After the quantification, the membranes were subjected to a standard stripping process, blocked again with the milk-supplemented TBS-T, and incubated overnight at 4 °C with rabbit polyclonal anti-GAPDH antibody (G9545, Sigma-Aldrich, St. Louis, MO, USA) diluted 1:10,000 with TBS-T. Next, the membranes were incubated for 1 h at room temperature with goat anti-rabbit IgG antibody–conjugated horseradish peroxidase conjugate (dil. 1:8000 with TBS-T supplemented with 5% (w/v) non-fat milk powder). GAPDH-containing immune complexes were visualized on an autoradiographic Hyperfilm-Kodak (Sigma-Aldrich, St. Louis, MO, USA) using an ECL kit (Thermo Fisher Scientific Inc. Rockford, IL, USA). The GAPDH bands were quantified as above. The contents of cytokines, iNOS, MOG, and CNPase were normalized to the contents of GAPDH (see Table 1).

### Determination of IL-10 concentration

IL-10 was assayed using a commercially available Rat Quantikine ELISA kit (R&D Systems Inc, Minneapolis, MN, USA) in lysate obtained from the brain cortex according to the manufacturer’s instructions.

### Determination of NF-κB activity in nuclear extracts from the brain cortex of EAE rats

Nuclear factor (NF)-κB activity was determined using a commercially-available ELISA kit (No. 10007889, Cayman Chemical Company, Ann Arbor, MI, USA) in a nuclear extract obtained from the brain cortex according to the manufacturer’s instructions.

### Determination of thiobarbituric acid reactive substances

Thiobarbituric acid reactive substances (TBARS), including malondialdehyde, which is the last product of lipid peroxidation, were determined according to Asakawa and Matsushita (1980). The homogenate was mixed with 10-mM Tris buffer (pH 7.4) at a protein concentration of approximately 1 mg/ml and incubated for 5 min. After incubation with 1 ml of 30% trichloric acid, 0.1 ml of 5-N HCl was added and centrifuged for 10 min at 4000×*g*; the supernatant was then collected, and 1 ml of 0.75% thiobarbituric acid was added. The tubes were capped and the heated at 100 °C for 15 min in a boiling water bath. Then, the optic density of the supernatant was determined at 535 nm in a Shimadzu UV 1202 spectrophotometer (Tokyo, Japan). TBARS concentration was calculated based on a standard curve obtained with a series of 1,1,3,3-tetraethoxypropane solutions.

### Statistical analysis

The results are expressed as the mean ±SEM. The differences between groups were analyzed using one-way ANOVA, followed by the Student–Newman–Keuls test when appropriate. *p* < 0.05 was considered significant.

## Results

### The effects of IFN-β1a and IFN-β1b treatment on the course of EAE

**Fig. 1:**
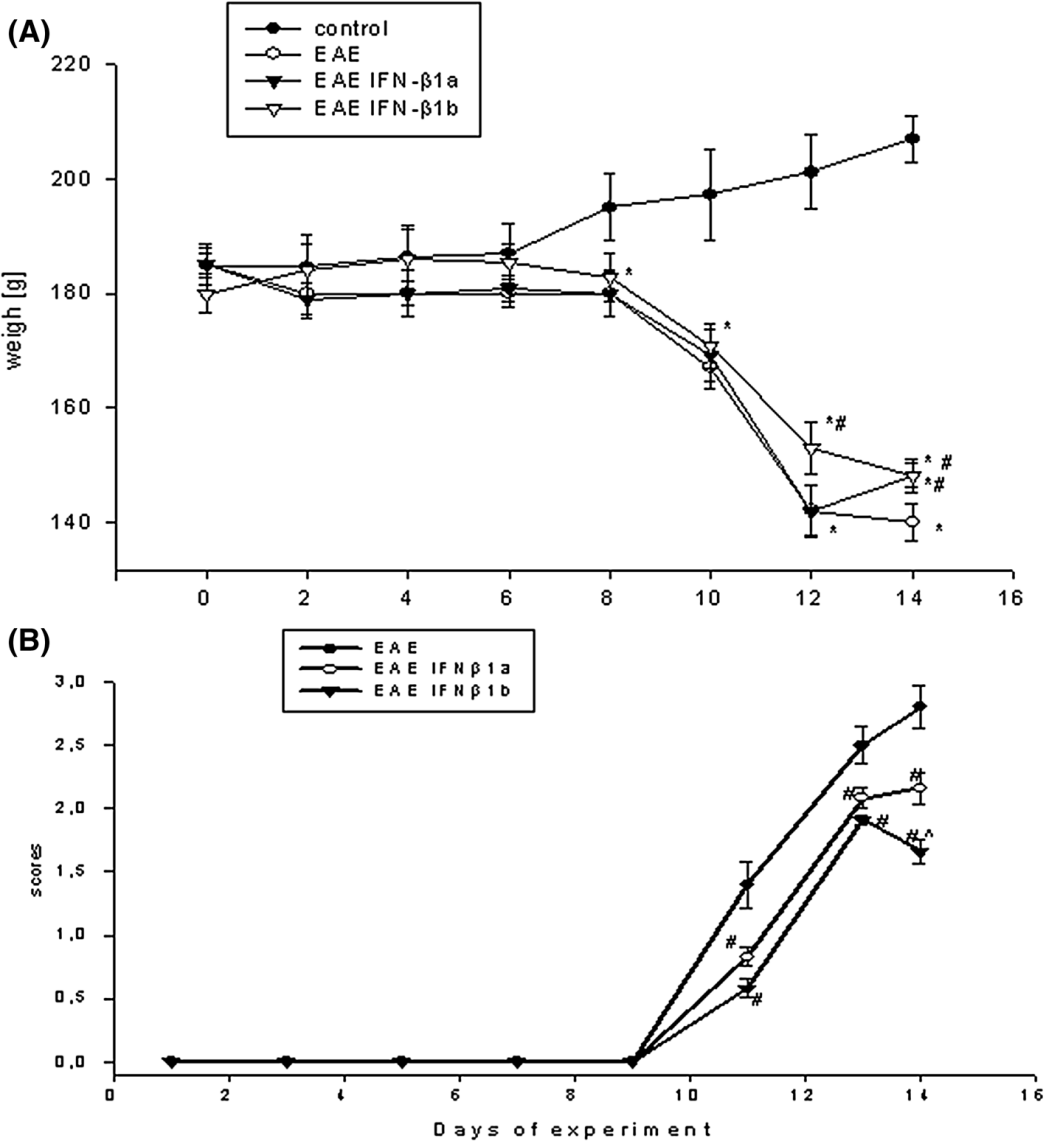
Weights of rats (**a**) and scores of the neurological symptoms (**b**) during the acute phase of EAE and after treatment with IFN-β1a and IFN-β1b. The results are the means ± SEM of data from 15 rats per group. *p < 0.05 versus control; ^#^ *p* < 0.05 versus EAE; ^^^ *p* < 0.05 versus EAE treated IFN-β1a (one-way ANOVA followed by the Student–Newman–Keuls test)

After inoculation, the neurological signs and body weight were measured daily. The neurological states were determined according to a scale from 0 to 5, as described in the Materials and Methods section. The first observed symptom of the disease, which was the loss of body weight, started around the 9th day post-immunization (d.p.i.) and progressed to the near end of the experiment, 13–14 d.p.i. (Sulkowski et al. 2013). During this phase of the disease, the rats lost approximately 20–25% of their body weight (Fig. 1a). The body weight started to significantly increase in the EAE rats treated with IFN-β1b after the 13th d.p.i. (*p* = 0.034; Fig. 1a). The body weights were approximately 10% greater in the EAE rats treated with both isoforms of IFN-β (IFN-β1a and IFN-β1b) compared with the untreated EAE rats at 14th d.p.i. (*p* = 0.041; Fig. 1a).

The neurological signs of EAE were demonstrated as a progressive developmental paralysis of the tail and the hind limbs and a reduction of physical activity. These signs of EAE started on the 9th d.p.i. and peaked on the 14th d.p.i. The EAE rats treated with the isoforms of IFN-β started to develop paralysis and peaked at the same d.p.i. as the EAE rats not treated with isoforms IFN-β. It was observed positive effects on the neurological deficits and an improved condition of the EAE rats following treatment with IFN-β1a and IFN-β1b at the 13th and 14th d.p.i. (*p* = 0.021; Fig. 1b). Moreover, EAE rats given IFN-β1b had a larger improvement in the neurological deficits and condition at 14th d.p.i., compared with those given IFN-β1a (*p* = 0.036; Fig. 1b).

### The effects of IFN-β1a and IFN-β1b treatment on astrocyte activation in the EAE rats

**Fig. 2:**
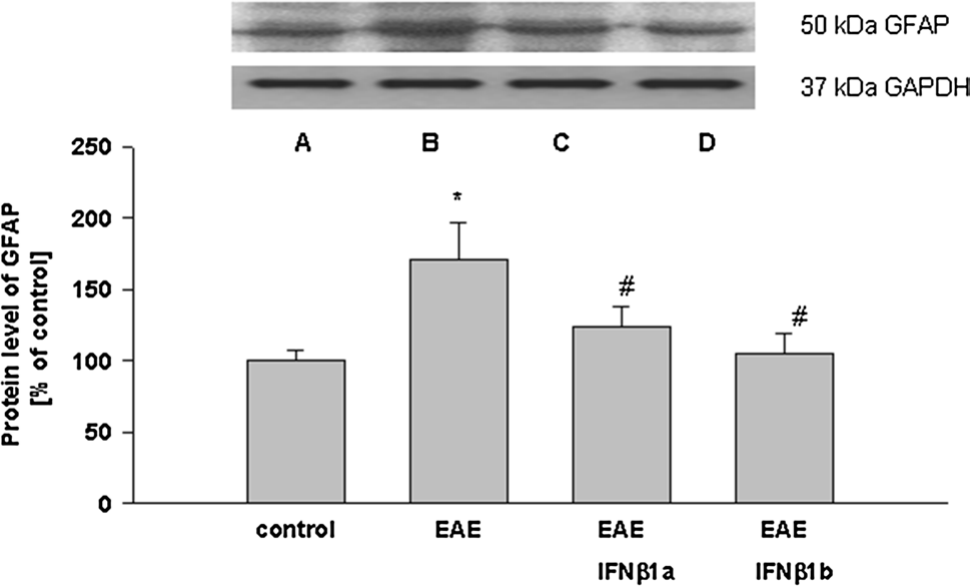
The effects of IFN-β1a and IFN-β1b on GFAP protein levels in the brain cortex in the acute phase of EAE rats. The images show Western blot analyses representative of 5 separate experiments. The results of densitometric analysis are shown as the mean ± SEM from 5 independent experiments and are expressed as the percentage of the control. The GFAP protein level was normalized to β-actin. *p < 0.05 versus the control; ^#^ *p* < 0.05 versus EAE (one-way ANOVA followed by the Student–Newman–Keuls test). *A* control, *B* EAE, *C* EAE IFN-β1a, *D* EAE IFN-β1b

In our study, the activation of astrocytes was observed in the brain cortex in the EAE rats compared with the control group (healthy rats) (Fig. 2). The GFAP protein level increased 1.7-fold in the brain cortex of the EAE rats compared to the control groups (*p* = 0.027). Both isoforms of IFN-β (IFN-β1a and IFN-β1b) significantly decreased the GFAP protein level in the brain cortex of the EAE rats compared with the untreated EAE rats (*p* = 0.031; Fig. 2).

### Effects of IFN-β1a and IFN-β1b treatment on cytokine expression in the brain cortices of the EAE rats

The analyses of the expression of pro- and anti-inflammation cytokines (mRNA and protein levels) were conducted in the peak stage of the disease (14th d.p.i.). It was observed that drastically increased pro- and anti-inflammation cytokines in the EAE rats at the peak stage of the disease (14th d.p.i.), which reached values from more than 1.4 to 12 times higher than the control (healthy) rats.

**Fig. 3:**
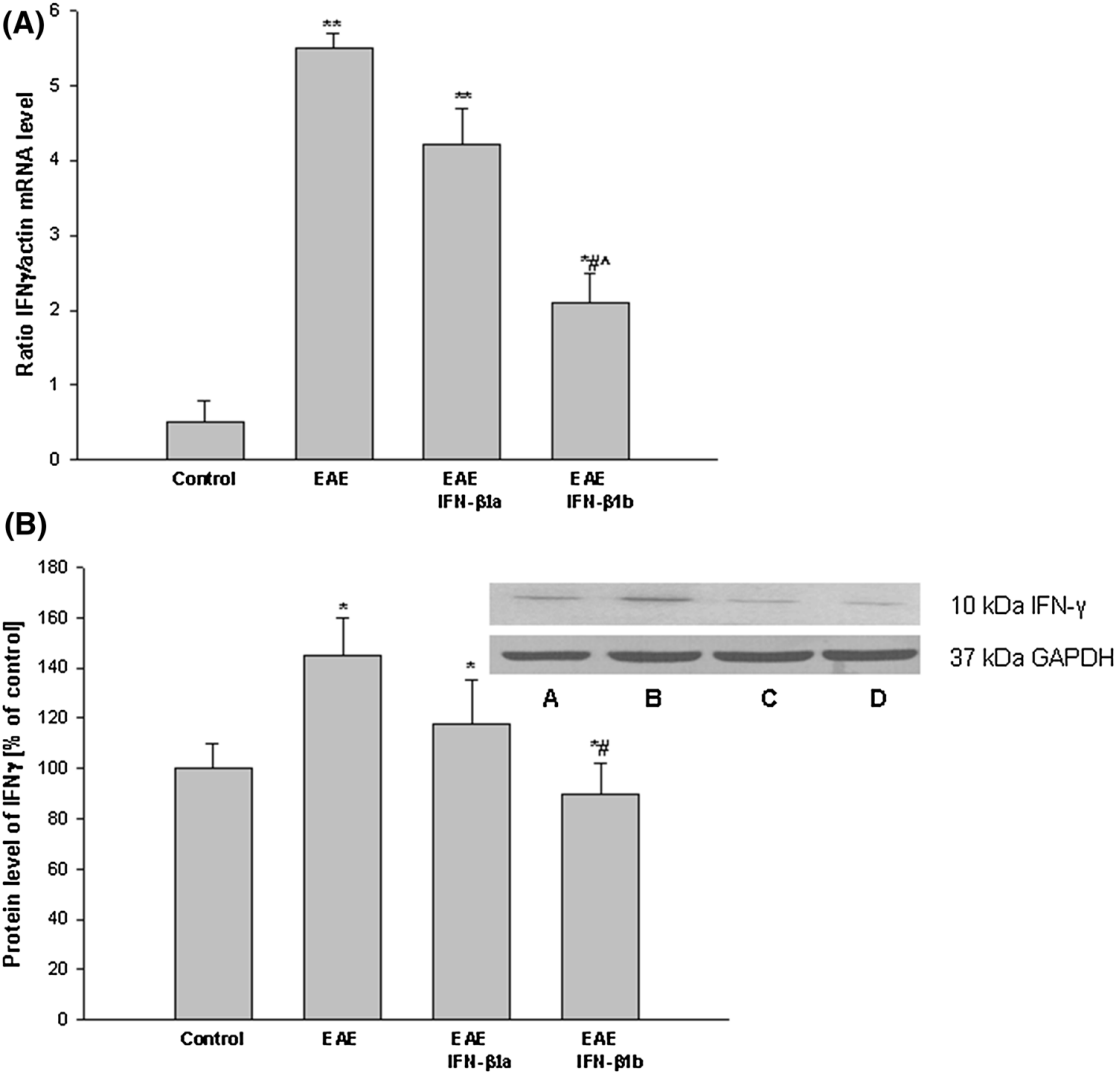
The effects of IFN-β1a and IFN-β1b on mRNA (**a**) and protein (**b**) levels of IFN-γ in the brain cortex in EAE rats. **a** The IFN-γ mRNA level was determined by quantitative real time-PCR (see “Materials and Methods”) and normalized against actin. The results are shown as the mean ± SEM of data from 6 independent experiments, each carried out in triplicate. **b** The images show the Western blot analyses representative of 5 separate experiments. The results of the densitometric analysis are shown as the mean ± SEM from 5 independent experiments and are expressed as the percentage of the control. IFN-γ protein levels were normalized to GAPDH. *A* control; *B* EAE; *C* EAE IFN-β1a; *D* EAE IFN-β1b; *p < 0.05; **p < 0.01 versus the control; ^#^ *p* < 0.05 versus EAE; ^^^ *p* < 0.05 versus EAE treated IFN-β1a (one-way ANOVA followed by the Student–Newman–Keuls test)

The level of IFN-γ mRNA drastically increased in the EAE rats at the peak stage of the disease (14th d.p.i.) and reached values more than five times higher than the control (healthy) rats (*p* = 0.0092; Fig. 3a). After the administration of IFN-β1b, the level of IFN-γ mRNA decreased by approximately 60% compared with the EAE untreated rats (*p* = 0.041; Fig. 3a). The smaller changes after the IFN-β1b treatment were observed in the case of the IFN-γ protein level, which decreased approximately 30% compared with the EAE untreated rats (*p* = 0.039; Fig. 3b). The treatment of the EAE rats with IFN-β1a did not change either the mRNA or the protein level of IFN-γ (Fig. 3a, b).

**Fig. 4:**
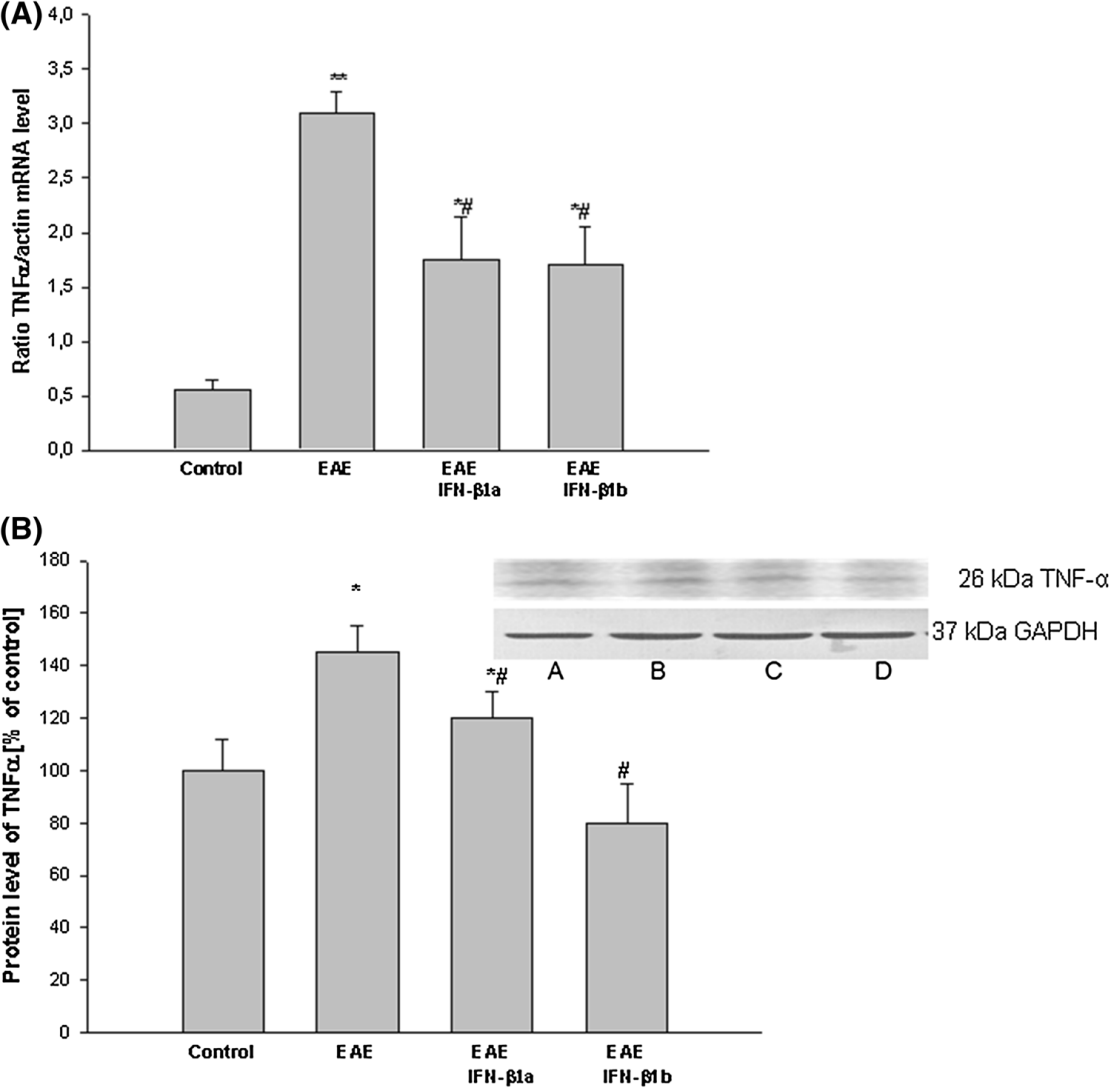
The effects of IFN-β1a and IFN-β1b on mRNA (**a**) and protein (**b**) levels of TNF-α in the brain cortex in EAE rats. **a** The mRNA TNF-α level was determined by quantitative real time-PCR (see “Materials and Methods”) and normalized against actin. The results are shown as the mean ± SEM of data from 6 independent experiments, each carried out in triplicate. **b** The images show Western blot analyses representative of 5 separate experiments. The results of the densitometric analysis are shown as the mean ± SEM from 5 independent experiments and are expressed as the percentage of the control. TNF-α protein levels were normalized to GAPDH. *A* control; *B* EAE; *C* EAE IFN-β1a; *D* EAE IFN-β1b; *p < 0.05; **p < 0.01 versus the control; #*p* < 0.05 versus EAE (one-way ANOVA followed by the Student–Newman–Keuls test)

The enhancement of the mRNA level of TNF-α in the EAE rats was then confirmed by Western blot analysis of the brain cortex homogenates (*p* = 0.045). These showed an increase in the levels of TNF-α mRNA and protein in the EAE rats at the 14th d.p.i. compared with the healthy control rats (*p* = 0.042; Fig. 4a, b). After treatment with IFN-β1a and IFN-β1b, the mRNA and protein levels of TNF-α decreased by approximately 50% compared with the untreated EAE rats. However, it remained approximately 50% above the control values (*p* = 0.037; Fig. 4a, b).

**Fig. 5:**
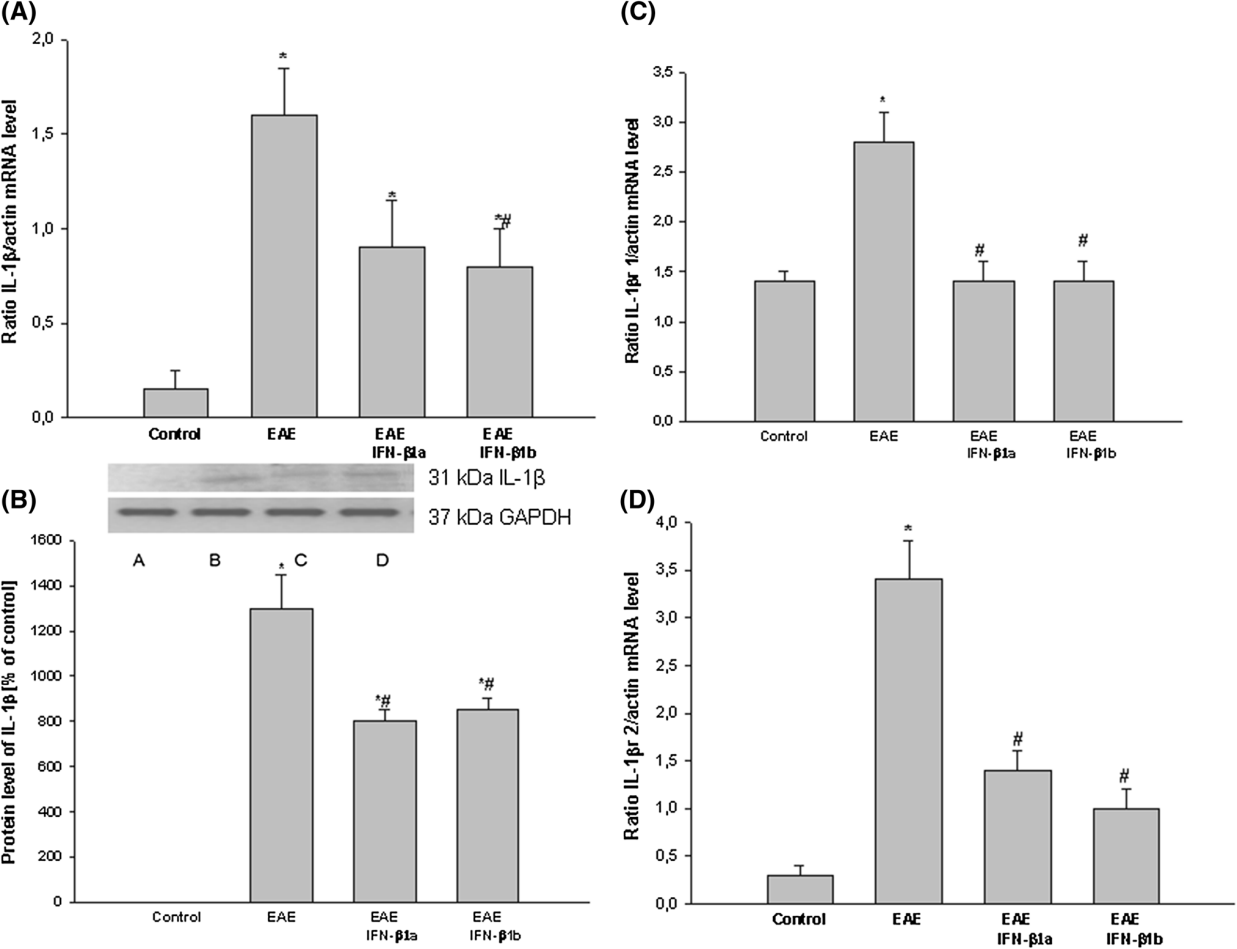
The effects of IFN-β1a and IFN-β1b on IL1β mRNA (**a**) and protein levels (**b**) and IL-1βR1 (**c**) and IL-1βR2 (**d**) mRNA level in the brain cortex in EAE rats. **a** The IL-1β, IL-1βR1 and IL-1βR2 mRNA mRNA levels were determined by quantitative real time-PCR (see “Materials and Methods”) and normalized against actin. The results are shown as the mean ± SEM of data from 6 independent experiments, each carried out in triplicate. **b** The images show Western blot analyses representative of 5 separate experiments. The results of the densitometric analysis are the mean ± SEM from 5 independent experiments and are expressed as the percentage of control. IL-1β protein levels were normalized to GAPDH. *A* control; *B* EAE; *C* EAE IFN-β1a, *D* EAE IFN-β1b; *p < 0.05 versus the control value; ^#^ *p* < 0.05 versus EAE (one-way ANOVA followed by the Student–Newman–Keuls test)

The level of IL-1β mRNA and protein drastically increased in the EAE rats at the peak of the disease (14 d.p.i.) and reached values more than 12 times higher than the control (healthy) rats (*p* = 0.029; Fig. 5a, b). After the administration of IFN-β1a and IFN-β1b, the levels decreased by approximately 45% compared with the EAE untreated rats (*p* = 0.026; Fig. 5a, b).

The levels of mRNA of IL-1β receptors R1 and R2 drastically increased in the EAE rats at the peak (14 d.p.i.) and reached values more than 6 times higher than the control (healthy) rats (*p* = 0.044; Fig. 5c, d). After the administration of IFN-β1a and IFN-β1b, the level of mRNA IL-1βR1 decreased to the control value (*p* = 0.039; Fig. 5c). The level of IL-1βR2 mRNA decreased by approximately 50% compared with the EAE untreated rats (*p* = 0.043; Fig. 5d).

**Fig. 6:**
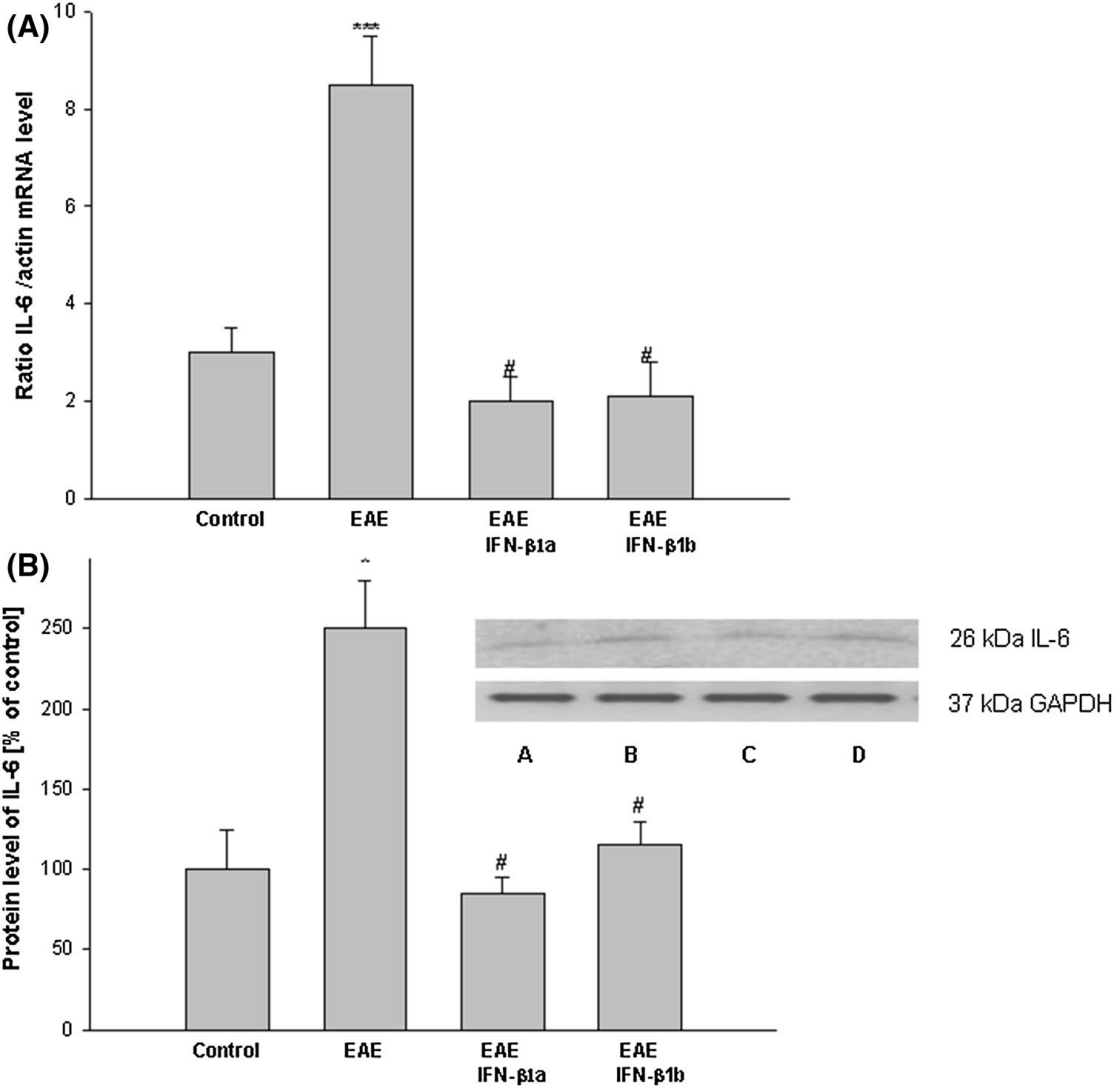
The effects of IFN-β1a and IFN-β1b on IL-6 mRNA (**a**) and protein (**b**) levels in the brain cortex in EAE rats. **a** The IL-6 mRNA level was determined by quantitative real time-PCR (see “Materials and Methods”) and normalized against actin. The results are shown as the mean ± SEM of data from 6 independent experiments, each carried out in triplicate. **b** The images show Western blot analyses representative of 5 separate experiments. The results of the densitometric analysis are the mean ± SEM from 5 independent experiments and are expressed as the percentage of the control. IL-6 protein levels were normalized to GAPDH. *A* control; *B* EAE; *C* EAE IFN-β1a; *D* EAE IFN-β1b; *p < 0.05, ***p < 0.001 versus the control; ^#^ *p* < 0.05 versus EAE (one-way ANOVA followed by the Student–Newman–Keuls test)

The level of mRNA and protein IL-6 increased 2.4-fold in the brain cortex of EAE rats compared with the controls (*p* = 0.037; Fig. 6a, b). The administration of IFN-β1a and IFN-β1b decreased the mRNA and protein levels of IL-6 in the brain cortex compared with the EAE untreated rats (*p* = 0.028; Fig. 6a, b).

**Fig. 7:**
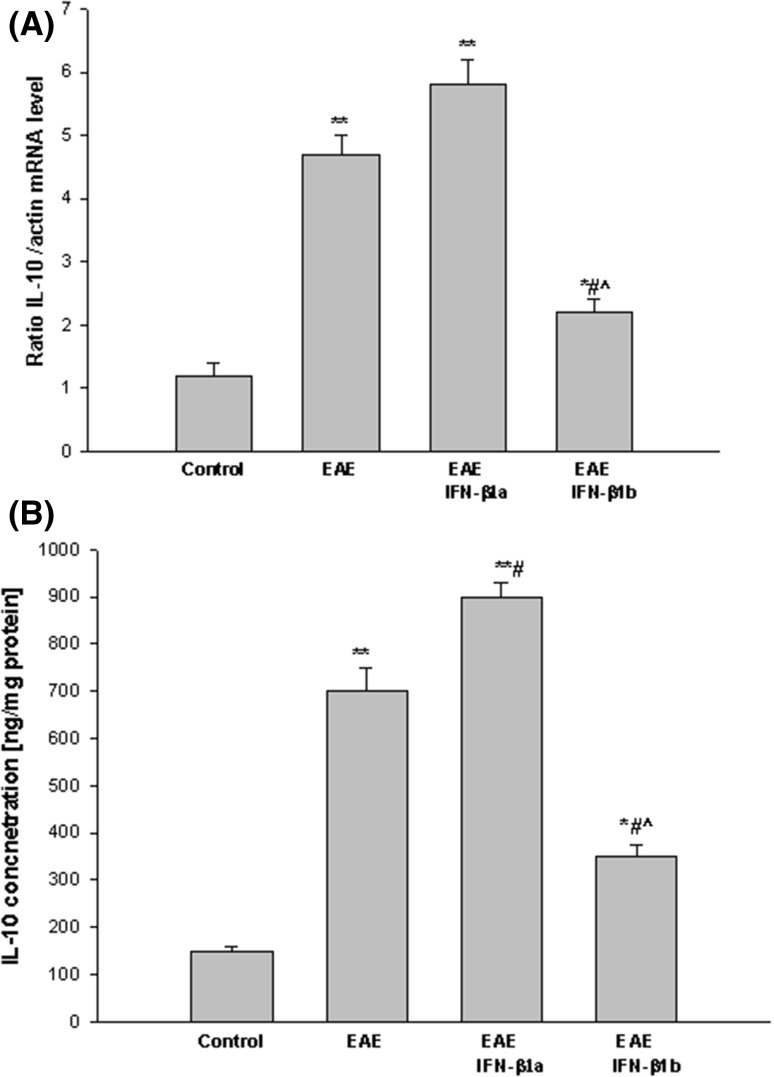
The effects of IFN-β1a and IFN-β1b on the IL-10 mRNA level in the brain cortex in EAE rats. The IL-10 mRNA level was determined by quantitative real time-PCR (see “Materials and Methods”) and normalized against actin. The results are shown as the mean ± SEM of data from 6 independent experiments, each carried out in triplicate. **b** The IL-10 concentration was determined by ELISA kit. The results are the mean ± SEM from 4 independent experiments in duplicated. *p < 0.05; **p < 0.01 versus the control value; #*p* < 0.01 versus EAE; ^^^ *p* < 0.05 versus EAE IFN-β1a (one-way ANOVA followed by the Student–Newman–Keuls test)

In the EAE rats, IL-10 mRNA and protein levels were significantly increased, about fourfold, in the brain cortex compared with the control group (*p* = 0.008; Fig. 7a, b). The subsequent increase in IL-10 mRNA and protein levels in the brain cortex of the EAE rats was seen after treatment with IFN-β1a as compared with the untreated EAE rats (*p* = 0.042; *p* < 0.05). Moreover, treatment with IFN-β1b decreased the levels in the brain cortex compared with the EAE untreated rats and reached values of more than two times higher than in the control (healthy) rats (*p* = 0.008; Fig. 7a, b).

### Effects of IFN-β1a and IFN-β1b treatment on pro-inflammatory mediators (NF-κB, iNOS, ROS) in the brain cortices of the EAE rats

**Fig. 8:**
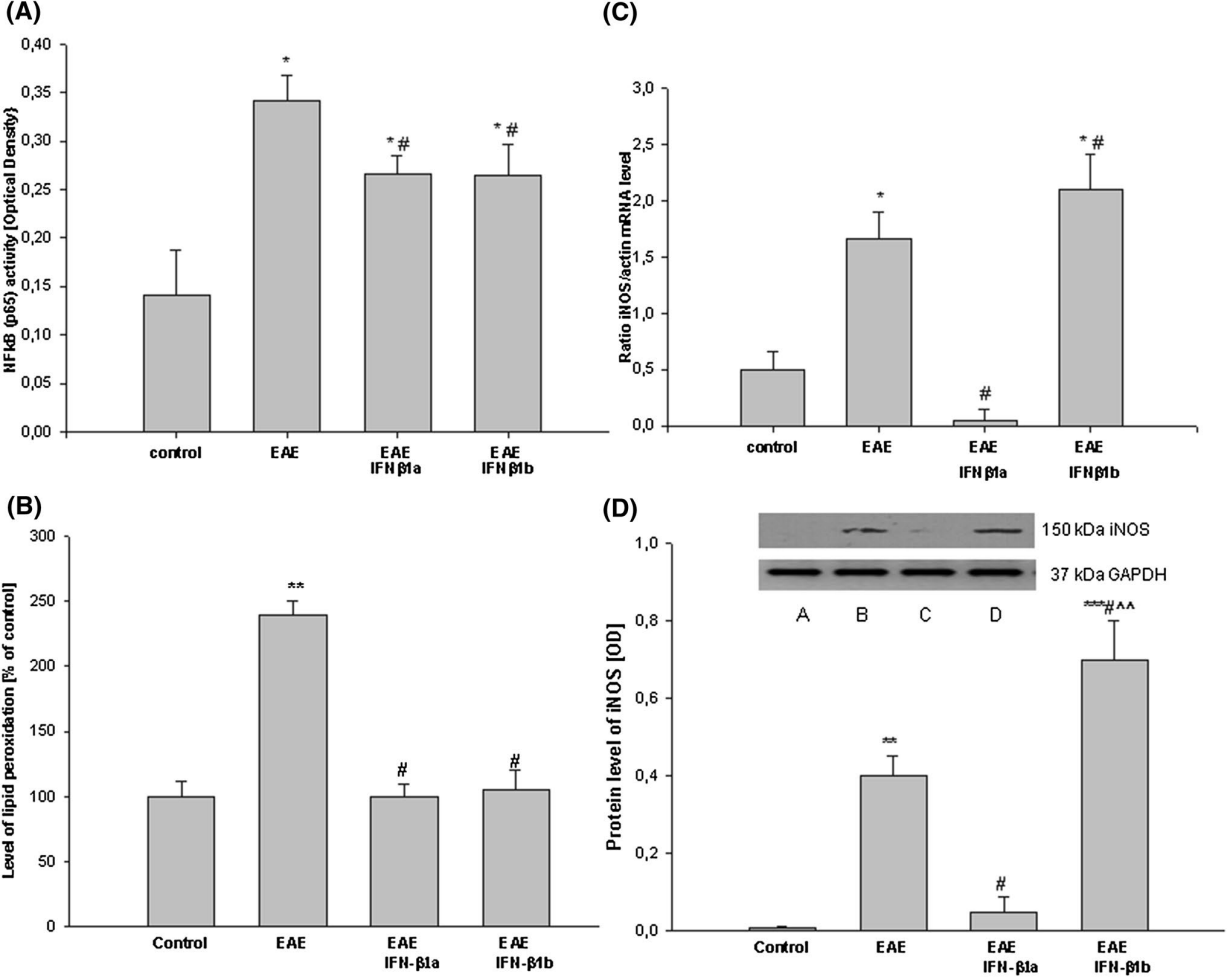
The effects of IFN-β1a and IFN-β1b on NF-κB (p65) activity (**a**), lipid peroxidation (**b**), mRNA (**c**) and protein level (**d**) iNOS in the brain cortex in EAE rats. **a** The NF-κB (p65) activity was determined applying a commercially-available ELISA kit to a nuclear extract obtained from the brain cortex according to the manufacturer’s instructions. **b** The iNOS mRNA level was determined by quantitative real time-PCR (see “Materials and Methods”) and normalized against actin. The results are shown as the mean ± SEM of data from 6 independent experiments. **d** The images show Western blot analyses representative of 5 separate experiments. The results of the densitometric analysis are the mean ± SEM from 5 independent experiments and are expressed as the percentage of the control. iNOS protein levels were normalized to GAPDH. *A* control; *B* EAE; *C* EAE IFN-β1a; *D* EAE IFN-β1b; *p < 0.05; **p < 0.01; ***p < 0.001 versus the control; ^#^ *p* < 0.01 versus EAE; ^^^^ *p* < 0.01 versus EAE treated with IFN-β1a (one-way ANOVA followed by the Student–Newman–Keuls test)

It was observed that EAE induced a 2.4-fold increase in NF-κB (p65) activity in the brain cortex compared with the control rats (*p* = 0.043; Fig. 8a). The treatment with both IFN-β1a and IFN-β1b decreased compared with the EAE untreated rats, but it was still more than 1.8 times higher compared with the control rats (*p* = 0.046; Fig. 8a).

It was observed increased lipid peroxidation in the brain cortex of the EAE rats compared with the control value (*p* = 0.0079; Fig. 8b). Both IFN-β1a and IFN-β1b inhibited significant (100%) lipid peroxidation in the cerebral cortex of the rats with EAE compared with the untreated EAE rats (*p* = 0.0074; Fig. 8b).

The increased pro-inflammatory cytokine (TNF-α, IFN-γ, IL-1β) levels and NF-κB activity were accompanied by the induction of iNOS in the brain cortex of the EAE rats at the peak stage of the disease (14th d.p.i.). The iNOS mRNA and protein levels were significantly enhanced, 3.3-fold, in the EAE rats compared with the control group (*p* = 0.029; Fig. 8c, d). The administration of IFN-β1a caused a significant decrease in iNOS expression in the brain cortex of the EAE rats, compared with the non-treated EAE value (*p* = 0.0085). Moreover, treatment with IFN-β1b did not decreased the EAE-induced iNOS mRNA and protein levels in the brain cortex of the EAE rats (Fig. 8c, d).

### The effects of treatment of IFN-β1a and IFN-β1b on myelin protein levels in the brain cortex of the EAE rats

**Fig. 9:**
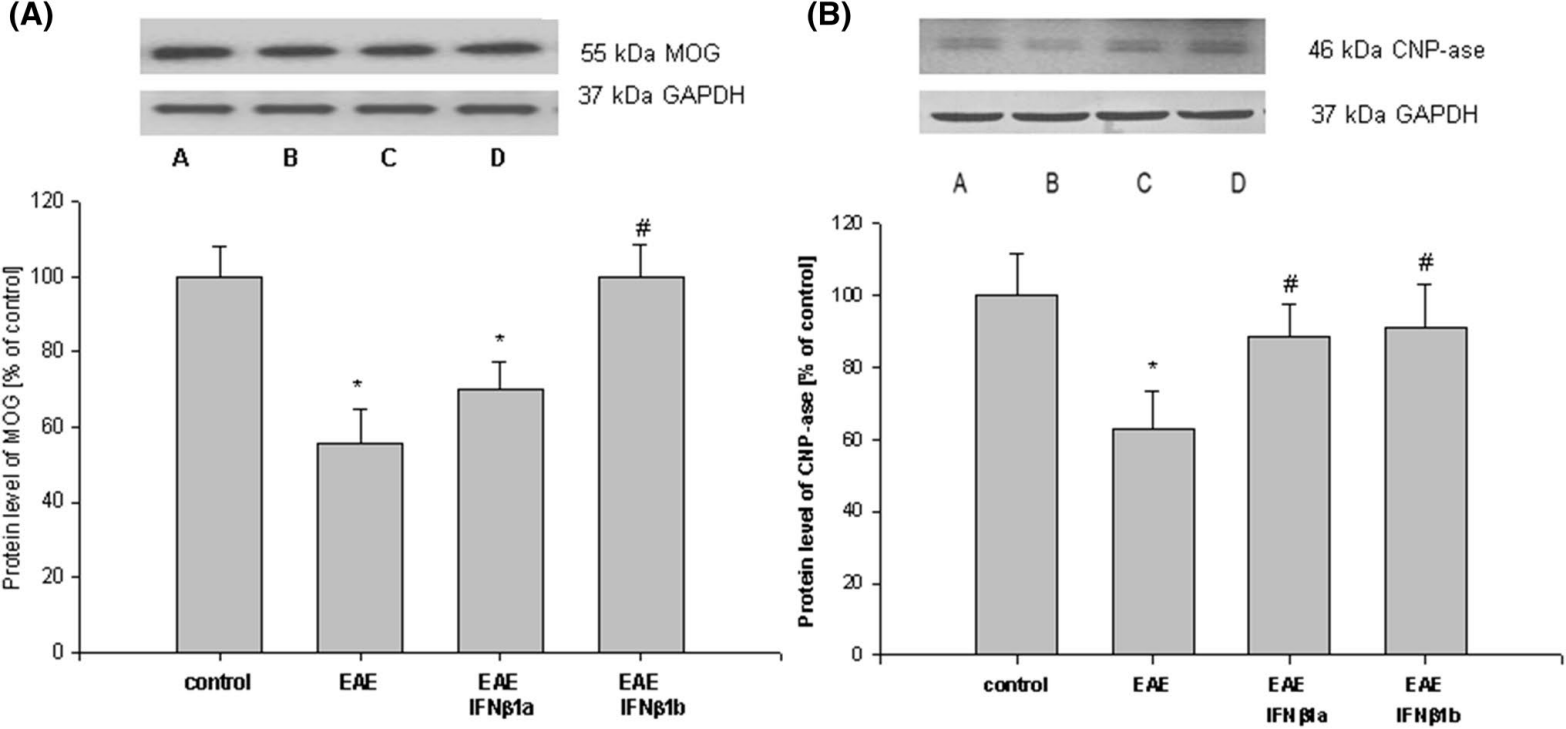
The effects of IFN-β1a and IFN-β1b on the CNP-ase and MOG protein level in the brain cortex in EAE rats. The images show Western blot analyses representative of 5 separate experiments. The results of the densitometric analysis are shown as the mean ± SEM from 5 independent experiments and are expressed as the percentage of the control. The CNP-ase and MOG protein levels were normalized to GAPDH. *A* control; *B* EAE; *C* EAE IFN-β1a; *D* EAE IFN-β1b; *p < 0.05 versus the control value; ^#^ *p* < 0.05 versus EAE (one-way ANOVA followed by the Student–Newman–Keuls test)

Western blot analysis showed a decrease in myelin protein (CNP-ase, MOG) levels in the brain cortex homogenates obtained from the EAE rats at 14th d.p.i. compared with the healthy rats (*p* = 0.028; Fig. 9). IFN-β1b caused a significant increase in the level of MOG protein compared with EAE values (*p* = 0.035, *p* < 0.05; Fig. 9a). In EAE rats treated with IFN-β1a, the MOG protein level was similar to that observed in the untreated EAE rats (*p* = 0.41; Fig. 9a).

The CNP-ase protein level was significantly decreased, approximately 40%, in the brain cortex of the EAE rats at the peak stage of the disease (14th d.p.i.) compared with the control rats (*p* = 0.036; Fig. 9b). The CNP-ase protein level increased to the control value in both groups of EAE rats treated with IFN-β1a or IFN-β1b (*p* = 0.061; Fig. 9b).

## Discussion

While the influence of IFN-β variants on the regulation of pro- and anti-inflammatory cytokines have been studied before (Graber 2007; Stępień et al. 2013), this study, to the best of our knowledge, is the first to investigate the effects of these factors in the early phase of the disease. By using an inoculums-induced EAE female rat model of MS, we provide evidence that both IFN-β variants improve neurological status, reduce pro-inflammatory cytokines and astrocyte activation as well as enhance MOG and CNP-ase myelin protein levels in early stage of disease. Despite the above general effects, it seems that the efficacy of used variants differs and the IFN-β1b had a higher protective effect than the IFN-β1a throughout the advance of EAE signs. It is clearly indicated by the complete recovery to control values of MOG and CNP-ase myelin protein levels by IFN-β1b and only partial recovery of MOG protein level by IFN-β1a treatments. It is likely that such an effect cannot be attributed to antiviral IFN-β variants activities because as was shown in vitro, antiviral activity of IFN-β1a is higher than that of IFN-β1b (Antonetti et al. 2002). However, another finding of this study with potential relevance for understanding of the differences in efficacy between both variants of IFN-β is the fact that brain cortex IL-10 was remarkably diminished after IFN-β1b treatment, while no changes was seen in case of IFN-β1a administration. Interestingly, the aforementioned changes in brain IL-10 levels coexisted with parallel alteration in iNOS mRNA and protein levels. These observations raise the possibility that there was a link between iNOS and the recovery process of MOG and CNP-ase myelin protein levels after IFN-β1b therapy during early stage of EAE disease process. In agreement with a possible involvement of iNOS in modulation of myelin protein remodeling are data showing participation of NO/cGMP/PKG pathway in myelin restoration (remyelination) in SM patients during remitting phase of disease (Nunes et al. 2012; Raposo et al. 2014). This observation supports a view that the structural differences between IFN-β1a and IFN-β1b may be a direct impact on their efficacy by modulating response of immune system which is also in accordance with data obtained after long term IFN-β treatment MS patients (Stępień et al. 2013; Tiberio et al. 2005; Zivadinov et al. 2007) and indirectly by modulating NO/cGMP/PKG pathway (Guthikonda et al. 1998).

In our study, we started selected treatment with IFN-β1a or IFN-β1b on the 8 day after immunization and proceeded adopted therapy until 14 day of disease (peak of stage EAE). This was done based on previous experiments with EAE showing that the neurological deficits and clinical severity achieved the highest level between 12 and 14 days. Thus, this experimental protocol let us answer the question of whether noticed above signs are reduced in response to applied IFN-β variants. Our experimental trials revealed the stronger protective effect of IFN-β1b compared with IFN-β1a. It was achieved by different regulation of the concentration of pro-and anti-inflammatory cytokine expression in the brain cortex at the peak stage of the disease (14th d.p.i.), which reached values of more than 2–12 times higher compared with the control (healthy) rats. Although both IFN-β1b and IFN-β1a partially inhibited the pro-inflammatory cytokines (IL-6, IL-1β, TNF-α) in the brain cortex in the EAE rats, only IFN-β1b decreased the concentration of the anti-inflammatory cytokine IL-10 and IFN-γ, while IFN-β1a did the opposite (Fig. 9). A previous study showed that, among these cytokines, dominant roles in MS pathology have been attributed to TNF-α, IL-1β, IL-6, and IFN-γ, the accumulation of which is associated with MS signs (Brosnan and Raine 1996; Molina-Holgado et al. 2001; Popko et al. 1997). All of these cytokines can damage myelin and oligodendrocytes and consequently cause damage to the axons and the deaths of neurons (Groomet al. 2003). Among these cytokines, TNF-α may play a particular role in MS pathology through its myelotoxicity, demonstrated in cultures of mouse spinal cord tissue (Selmaj and Rain 1988), and by the partial suppression of demyelination by a monoclonal anti-TNF-α antibody (Stoll et al. 1993). It is known that demyelination, oligodendrocyte loss, axonal damage, and astrogliosis are the histological hallmarks of MS (Kapadia and Sakic 2011; Shin et al. 2012; Wekerle 2008) and EAE (Aharoni et al. 2011).

The present data indicate that the key factor in the induction and course of EAE was the interplay between multiple cytokines and iNOS (Willenberg et al. 1999). iNOS up-regulation requires the involvement of TNF-α, IL-6, IL-1β and IFN-γ, and the latter, which is strongly associated with MS symptoms, is one of the predominant factors in the induction of iNOS expression by macrophages (Van der Veen et al. 2003). Inflammatory response to both IFN-β variants reduced the concentration of all investigated pro-inflammatory cytokines by about 50%, but it was only sufficient for iNOS induction in rats treated with IFN-β1b. NO, which is not engaged in free radical reactions, was shown to be involved in remyelination, because both IFN-β variants reduced oxidative stress. The most likely mechanism, which also helped NO serve its protective function, is the decrease in IL-10. It was shown that the observed changes in this study can take place in activated microglial and astrocyte cells in the acute phase of EAE (Kanwar et al. 2004; Shin et al. 2012; Sulkowski et al. 2013).

The immunization caused a decrease in MOG and CNP-ase protein levels in the brain cortex in the acute phase (14 d.p.i.) of EAE, which was associated with increased pro-inflammatory cytokines, decreased lipid peroxidation, and the activation of astrocyte cells. The administration of IFN-β1b to the EAE rats prevented some of the decrease in MOG and CNP-ase protein levels in the brain cortex, while IFN-β1a revealed the same pattern only in the case of CNP-ase, compared with the EAE rats (Fig. 9). These data suggested that IFN-β1b was most effective in the remyelination process. Our study revealed the same impact on pro-inflammatory cytokines of both IFN-β variants which may suggest that the stronger protective effect stimulated by IFN-β1a is caused by its influence on iNOS expression/NO synthesis.

In this study, we found that the iNOS mRNA and protein levels were meaningly enhanced, in the EAE rats compared with the control group (Fig. 8). These results are in agreement with several recent studies reporting up-regulation of iNOS (Calabrese et al. 2002; Kahl et al. 2003) and elevation of serum and CSF levels of its metabolites NO_3_ and NO_2_ in MS patients (Cross et al. 2006; Giovannoni et al. 1997; Johnson et al. 1995; Nazliel et al. 2002; Stepień et al. 2013). Although the knowledge of NO’s influence in the etiology and pathophysiology of MS is modest, the prevailing view is that iNOS reflects an inflammatory process coupled with activation macrophages/microglia and astrocytes leading to the tissue damage (Aguzzi et al. 2013; Okuda et al. 1995; Rieckmann et al. 1995). Besides the iNOS metabolites, NO may act directly as a reactive radical and indirectly by generation peroxynitrite anions (ONOO) (Beckman et al. 1994; Marques et al. 2008; Pautz et al. 2010) and also by its highly cytotoxic end product hydroxyl radical (^•^OH) (Beckman et al. 1994). All of them promote lipid peroxidation, the nitration of tyrosine residues on proteins and DNA, leading to their damage (Hoang et al. 2009). However, some controversies about the toxicity of NO in EAE/MS has been delivered by studies using NOS inhibitors. While administration of aminoguanidine (the more potent inhibitor of iNOS) was able to prevent the clinical symptoms of the disease in Swiss Jim Lambert mice (Brenner et al. 1997; Cross et al. 1994; Ljubisavljevic et al. 2011), others studies showed no significant therapeutic effects after administration of different NOS inhibitors to EAE rats (Ruuls et al. 1996; Zielasek et al. 1992).

We found that both isoforms of IFN-β, IFN-β1a and IFN-β1b, significantly decreased the GFAP protein level in the brain cortex in the EAE rats, compared with the untreated EAE rats. However, only IFN-β1b inhibited the immunization-induced enhancement of GFAP protein levels to the control values, which again indicates a more pronounced protective effect of IFN-β1b than of IFN-β1a. All said, it may point to the conclusion, that microglia and astrocytes, which are known to release potentially cytotoxic molecules, such as pro-inflammatory cytokines and reactive oxygen intermediates (Dheen et al. 2007), may be effectively influenced by IFN-β1b treatment.

In summary, our results indicated that both IFN-β1b and IFN-β1a treatment decreased pro-inflammatory cytokine (IL-6, IL-1β, TNF-α and IFN-γ) concentrations, microglia/astrocyte activation and oxidative stress in the brain cortex of the rats with EAE. Both IFN-β1b and IFN-β1a treatment improved the neurological status of the EAE rats and increased the myelin protein levels in the brain; however, IFN-β1b indicated a stronger protective effect on the inflammatory conditions in the brain compared with IFN-β1a. Differences in the improvements of the neurological status after treatment with IFN-β isoforms may result from the different influences on the levels of IL-10 and iNOS expression in (the peak of stage EAE) the brain cortex of EAE rats.
